# The Closed-Loop System Improved the Control of a Pregnant Patient with Type 1 Diabetes Mellitus

**DOI:** 10.1155/2021/7310176

**Published:** 2021-09-21

**Authors:** Guillermo E. Guzmán Gómez, Julian A. Viggiano, A. Silva-De Las Salas, Veline Martínez, María A. Urbano Bonilla

**Affiliations:** ^1^Fundación Clínica Valle del Lili, Department of Endocrinology, Universidad Icesi, Cali, Colombia; ^2^PGY3 Internal Medicine Resident, Fundación Clínica Valle del Lili, Universidad Icesi, Cali, Colombia; ^3^Universidad Icesi, Cali, Colombia; ^4^Fundación Clínica Valle del Lili, Department of Internal Medicine, Universidad Icesi, Cali, Colombia; ^5^Fundación Clínica Valle del Lili, Clinical Investigations Center, Cali, Colombia

## Abstract

**Objective:**

Closed-loop insulin systems represent a technological advance in diabetes management but have rarely been studied in pregnancy. We report a case of a patient with type 1 diabetes mellitus who was previously a user of the Paradigm VEO pump and then migrated to Medtronic 670G. *Research Design and Methods*. We reviewed the case of a G1P0 patient with type 1 diabetes, treated with the Medtronic 670G system during pregnancy; a comparison with current literature was done.

**Results:**

The patient achieved improved glycemic control as measured by time spent in different ranges as follows: <70 mg/dL, 8–4% and 70–180 mg/dL, 83–94%. Secondary outcomes included reduction of stress, anxiety, fear of hypoglycemia, and the psychological burden related to the disease. There were no obstetric or neonatal complications.

**Conclusion:**

The Medtronic 670G closed-loop system was used safely in a pregnant woman; nevertheless, further research is needed to validate this system in this patient population.

## 1. Introduction

Type 1 diabetes mellitus (DM1) increases the risk of obstetric and neonatal complications [1]. During gestation, hormonal changes cause a diabetogenic physiological state which can lead to volatile glycemic control in diabetic patients [2]. Previous studies have shown that DM1 women are up to five times more likely to experience severe hypoglycemia during early pregnancy compared to the preconception period [3]; the management of these patients can be challenging, and there is a need for new therapies. Advances in biotechnology seek to achieve glycemic control and minimize the burden of hypoglycemia.

The Medtronic MiniMed 670G is a closed-loop hybrid insulin infusion system approved for patients with diabetes [4, 5]; however, safety in pregnancy has not been established through clinical trials. One of the challenges with this pump is that its algorithm targets fasting glucose of 120 mg/dL, higher than the standard target of <95 mg/dL during pregnancy [6–10]. Herein, we present the case of a female patient with DM1 who achieved satisfactory glycemic control using the MiniMed 670G pump.

## 2. Methods

A 33-year-old woman with a history of DM1 since 16 years, user of an insulin pump (Paradigm® Veo system) due to high glycemic variability and recurrent hypoglycemia, presented to the outpatient clinic seeking preconception counseling. She reported no hypoglycemic episodes since 2015 and no macrovascular or microvascular complications.

She subsequently reported her pregnancy status at week 10. At the beginning of pregnancy, her weight was 57 Kg, body mass index (BMI) 23 Kg/m^2^, and glycated hemoglobin (HbA1c) 6.7%. At week 24, laboratories showed HbA1c of 6.3%; however, she developed high glycemic variability and inadvertent hypoglycemias. Enlite Sensor® continuous glucose monitoring (CGM) dysfunction was discovered, and a switch to a MiniMed 670G pump technology was decided.

During the following four weeks, the Paradigm® Veo pump was kept without the use of a sensor, precipitating hypoglycemia with self-reported anxiety and fear of a drop in glucose levels. At week 28, maternal weight was 63 Kg, and the estimated fetal weight was 1230 g for a 67th percentile (P67). At week 30, the training process on MiniMed 670 G began, a protocol that started with two weeks in the manual mode and was then switched to the automatic mode as an off-label indication. This optimized the times in range and reduced the hypoglycemic episodes and hence the related anxiety in the patient.

By week 33, the estimated fetal weight was 2291 g (P88) and at week 35 was 2832 g (P95) with a maternal weight of 65 Kg. The glycemic control ranges remained within goal until the end of the pregnancy, which took place at week 38 via cesarean section, during which the automatic mode and dextrose infusion were maintained (Table 1). The glycemic variability was higher in weeks 30–34 with higher time in range in hyperglycemia and hypoglycemia.

There were no obstetric complications, and a healthy female newborn weighting 3635 g, suitable for gestational age (P80), was born. In the immediate postpartum period, the automatic mode was suspended due to recurrent hypoglycemic episodes and the periodic suspension of the system by the patient. The manual mode was continued for four weeks, requiring weekly adjustments due to hypoglycemia and a significant decrease in the total required daily insulin dose. At the end of the fourth postpartum week, the automatic mode was restarted, and good glycemic control was maintained.

## 3. Discussion

DM1 increases the risk of obstetric and neonatal complications associated with maternal hyperglycemia in pregnancy such as macrosomia, congenital anomalies, shoulder dystocia, stillbirth, and neonatal death [1, 2, 11–13]. The glucose goals of pregnant patients are stricter: fasting and preprandial target glucose is <90 mg/dL. Therefore, various pharmacological management options have been developed with less variable insulin regimens that promote better glycemic control in pregnant women [14]. The insulin infusion pump (IIP) is a treatment strategy used when insulin regimens have not been able to achieve adequate control. This syste m has proven to be effective in reducing HbA1c in the nonpregnant population [15]. Along with the development of IIP systems, continuous glucose monitoring has emerged, and in combination, they improve clinical outcomes related to glycemic control. In pregnancy, the use of an insulin pump over multiple-dose injection therapy has not shown greater benefit [16–19]. However, the CONCEPTT trial demonstrated that the use of CGM helps improve neonatal outcomes and reduce maternal hyperglycemia [20].

Research has focused on the development of an artificial pancreas, which, through prediction algorithms, can increase or decrease the supply of insulin according to glucose levels obtained with CGM [21–24]. Stewart et al. compared a closed-loop system from a DANA Diabecare R insulin pump (SOOIL) and a CGM supported by FreeStyle Navigator II vs. an open-loop system in DM1 patients. Their findings reported that women under the closed-loop system achieved better glycemic control, evidenced by fewer obstetric and neonatal complications [25].

In 2016, the FDA approved the first hybrid system, the MiniMed 670G. This system differs from others since it has an automatic glycemic control algorithm that targets a nonmodifiable glucose goal of 120 mg/dL which is higher than the standard target for pregnant patients. The TIR International Consensus established that the target range for pregnant patients is 63–140 mg/dL and to stay below this range less than 5% of the time and above of it less than 25% of the time [26].

Given the short time, this system has been on the market, and there are few reports of its use during gestation. In the case of our patient, migrating from the VEO system to 670 G reduced hypoglycemia and increased time in the target glucose range at rates higher than what is reported in literature [27].

Additionally, the control of stress, anxiety, fear of hypoglycemia, and the psychological burden of the disease play an important role in the management of the diabetic patient. The use of CGM in the CONCEPTT trial showed a reduction in anxiety related to fear of hypoglycemia [20], improving the quality of life of patients using IIP systems coupled with CGM [28].

## 4. Conclusion

We report an adequate glycemic control in a pregnant patient with DM1 who was transitioned to a closed-loop system at 30 weeks of gestation. The patient had no macrovascular or microvascular complications. Patient showed decreased episodes of severe hypoglycemia and hyperglycemia with better glycemic control than previously reported cases. Our findings are encouraging and open the door for further clinical studies on the use of closed-loop systems in pregnancy.

## Figures and Tables

**Table 1:** Glycemic control monitoring.

Follow-up week	Pump	Mode	Time in range (TIR)				TDD (UI)	Basal/bolus ratio (%)
			<54 mg/dl (%)	54–63 mg/dl (%)	63–140 mg/dl (%)	>140 mg/dl		
30^*∗*^	VEO	N/A	1	4	87	8	40	38/62
32^*∗*^	670G	Manual	1	2	80	17	45	33/67
34^*∗*^	670G	Automatic	0	1	84	15	46	28/72
38^*∗*^	670G	Automatic	0	2	96	2	34	24/76
1^*∗∗*^	670G	Manual	1	2	94	3	29	21/79
4^*∗∗*^	670G	Manual	0	1	92	7	22	45/55
6^*∗∗*^	670G	Automatic	0	1	87	12	23	35/66

^*∗*^Follow-up during pregnancy. ^*∗∗*^Postpartum follow-up. TDD, total daily dose.
